# Use of an Electronic Clinical Decision Support System in Primary Care to Assess Inappropriate Polypharmacy in Young Seniors With Multimorbidity: Observational, Descriptive, Cross-Sectional Study

**DOI:** 10.2196/14130

**Published:** 2020-03-03

**Authors:** Eloisa Rogero-Blanco, Juan A Lopez-Rodriguez, Teresa Sanz-Cuesta, Mercedes Aza-Pascual-Salcedo, M Jose Bujalance-Zafra, Isabel Cura-Gonzalez, MultiPAP Group

**Affiliations:** 1 General Ricardos Primary Health Care Centre Madrid Spain; 2 Medical Specialties and Public Health School of Health Sciences University Rey Juan Carlos Alcorcón, Madrid Spain; 3 Health Services Research on Chronic Patients Network (REDISSEC) Madrid Spain; 4 Research Support Unit Primary Care Management Madrid Spain; 5 Dirección Farmacia Atención Primaria Sector Zaragoza III Zaragoza Spain; 6 Dirección Unidad Gestión Clínica Victoria en Málaga Servicio Andaluz de Salud Málaga Spain; 7 See Acknowledgments

**Keywords:** potentially inappropriate medication list, polypharmacy, multimorbidity, clinical decision support systems, primary care

## Abstract

**Background:**

Multimorbidity is a global health problem that is usually associated with polypharmacy, which increases the risk of potentially inappropriate prescribing (PIP). PIP entails higher hospitalization rates and mortality and increased usage of services provided by the health system. Tools exist to improve prescription practices and decrease PIP, including screening tools and explicit criteria that can be applied in an automated manner.

**Objective:**

This study aimed to describe the prevalence of PIP in primary care consultations among patients aged 65-75 years with multimorbidity and polypharmacy, detected by an electronic clinical decision support system (ECDSS) following the 2015 American Geriatrics Society Beers Criteria, the European Screening Tool of Older Person’s Prescription (STOPP), and the Screening Tool to Alert doctors to Right Treatment (START).

**Methods:**

This was an observational, descriptive, cross-sectional study. The sample included 593 community-dwelling adults aged 65-75 years (henceforth called young seniors), with multimorbidity (≥3 diseases) and polypharmacy (≥5 medications), who had visited their primary care doctor at least once over the last year at 1 of the 38 health care centers participating in the Multimorbidity and Polypharmacy in Primary Care (Multi-PAP) trial. Sociodemographic data, clinical and pharmacological treatment variables, and PIP, as detected by 1 ECDSS, were recorded. A multivariate logistic regression model with robust estimators was built to assess the factors affecting PIP according to the STOPP criteria.

**Results:**

PIP was detected in 57.0% (338/593; 95% CI 53-61) and 72.8% (432/593; 95% CI 69.3-76.4) of the patients according to the STOPP criteria and the Beers Criteria, respectively, whereas 42.8% (254/593; 95% CI 38.9-46.8) of the patients partially met the START criteria. The most frequently detected PIPs were benzodiazepines (BZD) intake for more than 4 weeks (217/593, 36.6%) using the STOPP version 2 and the prolonged use of proton pump inhibitors (269/593, 45.4%) using the 2015 Beers Criteria. Being a woman (odds ratio [OR] 1.43, 95% CI 1.01-2.01; *P*=.04), taking a greater number of medicines (OR 1.25, 95% CI 1.14-1.37; *P*<.04), working in the primary sector (OR 1.91, 95% CI 1.25-2.93; *P*=.003), and being prescribed drugs for the central nervous system (OR 3.75, 95% CI 2.45-5.76; *P*<.001) were related to a higher frequency of PIP.

**Conclusions:**

There is a high prevalence of PIP in primary care as detected by an ECDSS in community-dwelling young seniors with comorbidity and polypharmacy. The specific PIP criteria defined by this study are consistent with the current literature. This ECDSS can be useful for supervising prescriptions in primary health care consultations.

## Introduction

### Background

Multimorbidity is defined by the World Health Organization as the coexistence of two or more chronic illnesses in a person [1]. It is a growing phenomenon that has become a health problem and an international health challenge [2] resulting from increased life expectancy and nontransmittable disease rates, among other factors. Patients with multimorbidity usually present with polypharmacy, defined as the simultaneous usage of several medicines. This definition is controverted, and there is no standard agreement on the number of drugs that indicates that the patient is polymedicated [3].

Polypharmacy entails a greater risk for potentially inappropriate prescribing (PIP), which is defined as “the prescribing of medications or medication classes that should generally be avoided in persons 65 years or older because they are either ineffective or they pose unnecessarily high risk for older persons and a safer alternative is available (potentially inappropriate medication, PIM)” [4]. PIP also includes prescription omissions as the lack of use of potential right treatments. Data from the US National Health Survey yielded a prevalence of polypharmacy of 39% in the population aged older than 65 years [5]. A European study on medication dispensing for 310,000 adults within the UK National Health Service observed that the proportion of adult patients prescribed with 5 or more medicines doubled between 1995 and 2014, reaching 20.8% of the population [6]. For the same population age range in Spain, the percentage of polymedicated patients was approximately 50% [7]. The prevalence of patients with PIP is high, although it differs among studies, many of which are aimed at hospitalized, institutionalized, or immobilized elderly patients [8]. In the primary care setting in Europe, the PIP prevalence varies between 36% in Ireland [9] and 39% or 40.4% reported in 2 studies in Spain [10,11]. The existence of PIP has been related to a higher risk of being hospitalized [12], greater use of health emergency services and family doctor visits, reduced functionality [13], and increased mortality rates [14]. In terms of economic impact, some European studies estimate that the direct cost associated with PIP ranges between €188 and €318 (US $213-US $370) per patient per year [9,15].

A number of tools have been developed for studying appropriate prescribing and facilitating the detection of PIP. There are implicit methods based on clinical judgment, such as the Medication Appropriateness Index [16], and explicit methods based on predefined criteria, such as the American Geriatrics Society Beers Criteria, the European Screening Tool of Older Person’s Prescribing (STOPP) Criteria, and the Screening Tool to Alert Doctors to the Right Treatment (START) Criteria. Although the Beers Criteria were the first to be published in 1991 [4] and have been the most widely employed to date, their application in the European setting is limited because they include a high percentage of pharmacological drugs not recognized in the majority of catalogs for European countries. To address this limitation, the criteria for the STOPP and START were set in 2008. Studies comparing the STOPP Criteria with other explicit criteria show that the STOPP Criteria are more exhaustive [9,15,17]. The STOPP, START, and Beers Criteria were updated and validated in Spanish in 2015 [18,19], but there are no studies comparing them.

Some studies indicate that using the abovementioned tools to check prescribed medication translates into improved health outcomes for patients (falls, functionality, hospitalizations, number of consultations, and use of emergency services) [20]. However, applying these criteria requires extensive and updated clinical knowledge, time, and access to various simultaneous sources of information. For their use to be viable at primary care consultations, health professionals need automated, quick-to-consult tools that facilitate and supervise the process of medication prescribing. Electronic clinical decision support systems (ECDSSs) can improve prescription quality and reduce medication errors [21,22]. Although electronic clinical records are largely implemented in the Spanish primary care system, no adequate ECDSSs are currently available. Several clinical trials are being conducted to evaluate the use of ECDSSs in clinical practice [23-27].

In Spain, an available ECDSS (CheckTheMeds Technology SL) has been used in the hospital setting and in pharmacological services offering a personalized system for medication dispensing [28,29]. This Web-based tool globally processes each patient’s information, combining clinical and pharmacological data, thus offering the health professional an analysis of criteria (Beers, STOPP, and START) for detecting PIP in real time.

### Objective

The main aim of this study was to estimate the prevalence of PIP in young seniors (aged 65 to 75 years) with multimorbidity and polypharmacy, according to the updated criteria by the STOPP, START, and Beers, as detected by the ECDSS, at primary care consultations. The secondary objectives were to assess the relationships between clinical and sociodemographic variables and the presence of PIP.

## Methods

### Design

This was an observational, cross-sectional, descriptive, multicentric study conducted in the Spanish primary care setting.

### Population

This study included patients aged 65-75 years with multimorbidity (≥3 diseases) and polypharmacy (≥5 consumed medicines for at least 3 months), who had attended their doctor consultation at least once over the last year and provided written consent to participate in the Multimorbidity and Polypharmacy in Primary Care (Multi-PAP) trial [30]. Institutionalized patients—those whose life expectancy was less than 12 months, as estimated by their doctor—and patients with any severe mental disorder were excluded. Recruitment physicians did not receive any economic incentives. Each doctor offered participation to patients from a random list of patients potentially meeting the inclusion criteria. A total of 117 family doctors from 38 health care centers from 3 Spanish regions (Andalucia, Aragon, and Madrid) included a total of 593 subjects who agreed to participate. On the basis of the previous studies reporting a PIP percentage of 39% [10], with this sample size, a maximum type 1 error of 3.9% with a 95% CI was reported.

### Variables

The following sociodemographic variables were recorded for patients: age, gender, marital status, educational level, social class according to the Spanish classification [31], and family income in thousands of euros adjusted by the number of people in the household using the method proposed by the Organisation for Economic Cooperation and Development. In addition, the following clinical variables were collected: number of active pharmaceutical ingredients per patient according to the Anatomical Therapeutic Chemical (ATC) classification system and chronic conditions in accordance with the International Classification of Primary Care, with the most relevant ones selected according to the criterion by O’Halloran et al [32]. The assessed PIP variables were existence of STOPP and START criteria following its Spanish-adapted 2014 version [18], existence of Beers 2015 version criteria [19], number of STOPP criteria, number of START criteria, number of Beers Criteria, and classification of the type of detected criteria.

Sociodemographic and clinical data were obtained between December 2016 and January 2017 through an interview by each patient’s doctor and recorded in a data collection notebook (DCN). Subsequently, in April 2018, the data were uploaded from the DCN into the ECDSS. One researcher with vast clinical and therapeutic drug monitoring experience supervised the information transfer to the CheckTheMeds Technology SL and used this tool to globally review the treatment of all patients. The latest versions of the STOPP, START, and Beers Criteria were employed.

All the STOPP and Beers Criteria were analyzed. In agreement with previous studies [33] and to avoid potential information bias, this research team agreed on omitting the A1 STOPP criterion from the analysis (any drug prescribed but not indicated by clinical evidence) to prevent its overestimation [33].

### Statistical Analysis

Categorical variables were described by their frequencies and percentages. Quantitative variables were described by their mean and SD, with their corresponding 95% CI when they fit a normal distribution, or by their median and IQR in the case of asymmetric distributions. The presence of one or more PIP was identified, and the association between groups was assessed for the main variables using a chi-square test in the case of categorical variables or the Student *t* test (Mann-Whitney *U* test when the variable did not fit a normal distribution) in the case of quantitative ones. A multivariate logistic regression model was built to study factors related to PIP, with robust estimators that controlled for the effect of cluster sampling. The dependent variable was the presence of one or more PIP according to the STOPP version 2 (V2) criteria, and independent variables were those reaching statistical significance in the bivariate analysis or considered of clinical relevance. Stata version 14.0 (StataCorp LLC) and IBM SPSS 21 software (IBM Corp) were employed in the statistical analyses.

## Results

A total of 4386 prescriptions were recorded for the 593 included patients (593/635, 93.4% of the total offered for participation). The average age of patients was 69.7 (SD 2.7) years; 56.3% (334/593) of the patients were women, 75.4% (447/593) were married, and 17.9% (106/593) lived by themselves. Multimedia Appendix 1 describes the main sociodemographic and clinical characteristics for each group according to the presence or absence of PIP or medication omissions according to the START Criteria. The most frequent pathologies were high blood pressure that amounted to 78.9% cases (468/593) and hypercholesterolemia with 50.2% cases (301/593). Of the 593 patients, 250 (42.2%) were diabetic, 215 (36.3%) had arthritis (knee, hip, or other joints), and 220 (37.1%) had mental disorders (for a complete list of disease prevalence, see Multimedia Appendix 2). The median number of chronic illnesses per patient was 5 (IQR 4-7). The median number of medicines per patient was 7 (IQR 6-9), and 17.9% (106/593) of the patients were prescribed ≥10 drugs. In terms of ATC groups, the cardiovascular one was the most frequent, with 95.3% (565/593) subjects taking at least one drug, followed by the digestive system/metabolism and the nervous system groups. The most prescribed drug was omeprazole, which was taken by 49.2% (292/593) of patients, followed by acetylsalicylic acid by 36.8% (218/593) of patients, metformin by 34.4% (204/593) of patients, simvastatin by 32.9% (195/593) of patients, and enalapril by 27.2% (161/593) of patients.

The frequency for the ECDSS to detect at least one explicit criterion was 57.0% (95% CI 53-61), 42.8% (95% CI 38.9-46.8), and 72.8% (95% CI 69.3-76.4) applying the STOPP, START, and Beers Criteria, respectively, all in their latest versions at the point when this study was conducted (Table 1). The percentage of patients that met three or more Beers Criteria was 16.5% (98/593). Of the overall criteria evaluated for the studied sample, 30 different criteria were detected in the STOPP, 21 in the START, and 34 in the Beers Criteria (see Multimedia Appendix 3). The most frequently found PIP according to the STOPP criteria were the prolonged use of benzodiazepines (BZD) in 36.6% of patients (217/593), followed by beta blockers in 12.5% (74/593) of patients with diabetes mellitus with frequent episodes of hypoglycemia and the prescribed use of opioids without associated laxatives found in 5.4% (32/593) of patients. Following the Beers Criteria, the most frequent PIP were the prolonged use of proton pump inhibitors (PPIs) by 45.4% (269/593) of patients, prolonged use of BZD and hypnotic drugs by 27% (160/593) of patients, and prolonged use of antidepressants or the combined use of 2 or more central nervous system depressants by 17.4% (130/593) of patients (Multimedia Appendix 2).

At least one PIP was detected using both methods in 40.8% (242/593) of patients, and disagreement was found in 32.0% (190/593) of patients for whom 1 PIP was detected according to the Beers Criteria but did not meet any criterion in the STOPP. On the contrary, 16.2% (96/593) of patients presented with some PIP using the STOPP criteria, but none using the Beers Criteria.

Factors that were found to relate to the presence of PIP were being a woman, greater medicine intake, belonging to social class 2 (qualified primary sector), and using a pharmacological treatment for the central nervous system (Anatomical Therapeutic Chemical classification system-nervous system [ATC-N]; see Table 2).

The most frequent omission of medicines detected by the START criteria in 593 patients were inhaled corticosteroids in 55 (9.3%) patients with asthma or severe chronic obstructive pulmonary disease (COPD); prostaglandins, prostamide, or topical beta blockers in 40 (6.7%) patients with primary open-angle glaucoma; calcium and vitamin D supplements in 37 (6.2%) patients with osteoporosis; and inhibitors of 5α-reductase also in 37 (6.2%) patients for the treatment of symptomatic prostatism when prostatectomy is not necessary (see Multimedia Appendix 2).

**Table 1:** Percentage of detected potentially inappropriate prescribing (potentially inappropriate medication or prescription omission) according to the different criteria sets.

Potentially inappropriate prescribing			Screening Tool of Older Person’s potentially inappropriate prescriptions version 2				Beers 2015 version				Screening Tool to Alert doctors to Right Treatment, version 2		
			n (%)		95% CI		n (%)		95% CI		n (%)		95% CI
Patients with at least one PIM^a^ or one MO^b^			338 (57.0)		53-61		432 (72.8)		69.3-76.4		254 (42.0)		38.8-46.8
**Number of PIM/MO per patient**													
	1	257 (43.3)		N/A^c^		214 (36.1)		N/A		160 (27.0)		N/A	
	2	65 (11.0)		N/A		120 (20.2)		N/A		64 (10.8)		N/A	
	≥3	16 (2.7)		N/A		98 (16.5)		N/A		28 (4.7)		N/A	

^a^PIM: potentially inappropriate medication.

^b^MO: medication omission.

^c^N/A: not applicable.

**Table 2:** Factors associated with a potentially inappropriate medication according to the Screening Tool of Older Person’s Prescription criteria.

Associated factors			Odds ratio (95% CI)		*P* value
Woman			1.43 (1.01-2.01)		.04
Number of drugs			1.25 (1.14-1.37)		<.001
**Social class**					
	Unskilled	Reference		N/A^a^	
	Skilled worker in the primary sector	1.45 (0.98-2.16)		.07	
	Supervisors, managers, and directors	1.91 (1.25-2.93)		.003	
Usage of drugs in Anatomical Therapeutic Chemical classification system-nervous system group			3.75 (2.43-5.76)		<.001

^a^N/A: not applicable.

## Discussion

### Principal Findings

The percentage of patients in the sample with one or more PIP was 57.0% (338/593) using STOPP (95% CI 53-61), 72.8% (432/593) with Beers (95% CI 69.3-76.4), and 42.8% (254/593) with START (95% CI 38.9-46.8). The most frequent PIPs were the use of BZD for more than 4 weeks according to the STOPP V2 criteria (217/593, 36.6%) and the prolonged use of PPI using the Beers 2015 version (269/593, 45.4%). Factors associated with a higher PIP frequency were being a woman (OR 1.43, 95% CI 1.01-2.01; *P*=.04), greater medicine intake (OR 1.25, 95% CI 1.14-1.37; *P*<.001), being a primary sector worker (OR 1.91, 95% CI 1.25-2.93; *P*=.003), and using pharmacological treatments for the central nervous system (OR 3.75, 95% CI 2.45-5.76; *P*<.001). Both the STOPP and Beers tools simultaneously detected one or more PIP in 40.8% (242/593) of the patients. Differences were found using the STOPP and Beers sets of criteria, and the difficulties for using some of the explicit criteria were found by the ECDSS.

### Prevalence of Potentially Inappropriate Prescribing

The prevalence of PIP among community-dwelling young seniors varies depending on the explicit criteria employed as well as the different study designs and included populations [15,18,34]. This study obtained a prevalence of 57.0% in accordance with the STOPP criteria, which is higher than that of previous studies [9,10] using previous STOPP versions in the primary care setting and the European population (36% and 39%, respectively). Studies using the STOPP 2014 version found prevalence values of 8.7% and 40.4% [11,33]. The study by Blanco-Reina et al [11], with similar data to ours, obtained a prevalence of 40.4% in contrast to the 18.7% obtained using the original STOPP version and concluded that the latest version is more sensitive for PIP detection. The greater prevalence observed in this study can result from the included population, which had to meet the polypharmacy criterion to participate in the Multi-PAP trial group, whereas only 72.9% of the patients in the abovementioned study were polymedicated.

Following the Beers 2015 version criteria, the PIP prevalence (77.2%) was higher than that described in some previous studies. Owing to the repeated updates of these criteria (5 up to 2015) and different study designs and studied populations, there is great variability in the obtained prevalence values [34]. Studies using the Beers 2015 version reported a prevalence ranging from 53.5% in the hospitalized population [35] to 72.8% in studies based on big data [36]. In Spain, using the 2008 and 2012 versions, the prevalence of PIP varied from 24.3% to 44.0%, respectively [37].

Studies comparing the 2015 version with the 2012 versions of Beers Criteria [35] confirmed that the latest version has greater sensitivity for detecting PIP. The increased number of criteria in the Beers 2015 version and the adaptation of the medications catalog [17], together with the application of these criteria to a polymedicated population, can explain the higher prevalence found in our sample.

The percentage of patients with one or more medication omission was 42.8%, a figure superior to those in previous studies, where it ranged between 22.0% and 39.9% using the START criteria [10,11,33,37].

### Most Frequent Screening Tool of Older Person’s Prescription and Screening Tool to Alert Doctors to Right Treatment, the Beers Criteria

In agreement with most previous studies, BZD and PPI were the most frequent PIP detected [10,11,15,33,37]. The proportion of patients using BZD for more than 4 weeks in the studied sample using STOPP V2 is similar to that found by Blanco-Reina et al [11] (36.6% vs 38.6%, respectively), whereas the use of BZD and hypnotic drugs using the Beers Criteria was lower than that observed in the mentioned study (26.9% vs 52.4%, respectively) but similar to that in a study by Zhang et al (29.8%) [35]. The reason for this difference can be that the most used BZD by patients in this study were lorazepam (13.5%), bromazepam (8.6%), and lormetazepam (6.9%), with the 2 latest being included in the catalog of medicines detected by the STOPP criteria but not in the catalog for the Beers. The work by Blanco-Reina et al [11], which did not specify what the most used BZD were, reported that 46.7% of patients had insomnia, a figure much higher than the one observed in our sample, which could explain the differences between them.

This study found that the prolonged use of PPI was the most frequent PIP detected by the Beers latest version (45.5%), although it was not detected using STOPP. Several systematic reviews report it as one of the most frequent PIP observed in the polymedicated population in accordance with different explicit criteria [15,34,38]. The Beers 2015 updated version included this criterion that is already described as frequent (41.9%) by some authors [35], although their samples are not comparable with ours. Some studies that identified this PIP as frequent [9,10] employed a modified version of STOPP that facilitates its detection, instead of the original criterion that is associated with a specific clinical condition that limits its capacity for such purpose (PPI for treating peptic ulcer disease without complications or peptic erosive esophagitis with full therapeutic doses for more than 8 weeks). For example, 40 mg of omeprazole was considered the full therapeutic dose by the ECDSS.

The use of nonsteroidal anti-inflammatory drugs (NSAIDs) also appears in the current literature as one the most frequently found PIP in the polymedicated population [15,34,38]. This criterion only was met by 1.5% of patients in the study by Blanco-Reina et al [11], which used a similar methodology to ours. As their own family doctors were the ones entering the pharmacological information for their patients in the DCN, a bias can be present in the outcome if the patient was taking the NSAIDs, but the doctor did not consider it as their regular chronic medication and hence did not include it in the DCN. This record omission, together with the ample information on increased cardiovascular risk associated with these drugs, can explain the observed differences.

In studies using the START 2014, the most relevant omission criterion is the use of inhaled corticosteroids in patients with asthma or severe COPD as well as the use of calcium and vitamin D supplements in patients with osteoporosis, as was the case in our sample. The criterion for prostaglandin, prostamides, or topical beta blockers in patients with primary open-angle glaucoma can be overestimated because topical medication was not recorded, which can partially explain the high prevalence of omissions in this trial.

Factors relating to the presence of PIP as detected using the STOPP are similar to those in previous studies [15,34] that have identified a relationship between being a woman and taking a greater number of medicines with the presence of PIP. These studies also found a correlation with poor economic status, which was not the case in our study, although the classification systems for the economic level and social class are not comparable. The ATC-N includes all the drugs in the BZD group as well as antidepressants, thus its association with the presence of PIP.

### Agreement Between Methods

Only 40.0% (237/593) of patients were simultaneously detected with one or more PIP according to the criteria of the Beers and STOPP methods. The use of different medication catalogs by these tools does not entirely explain the observed discrepancies. Although previous studies have analyzed the agreement between both the tools in terms of the overall percentage of detected PIP [11,37,39], they did not take into consideration that the drugs and clinical scenarios assessed by each method are different and changed throughout their updated versions. In a review by Motter et al [17], a detailed analysis of differences among the existing explicit criteria since 1991 (STOPP, Beers, and others) was conducted; they concluded that these tools assess different parameters, medicines, and clinical scenarios, which are not readily comparable. Given the differences between these tools, their combined use in a complementary manner is probably the most adequate approach.

### Algorithms Used by the Electronic Clinical Decision Support System

ECDSSs are very helpful tools for applying different explicit criteria simultaneously [40,41]. Translating the STOPP criteria into computerized algorithms to be used by an ECDSS can be more complicated than doing so for the Beers Criteria. This was evident in our study in terms of the difficulty found in detecting the usage of PPIs for more than 8 weeks using the STOPP criteria, as previously mentioned. The majority of the STOPP criteria are linked to a clinical condition that is often difficult to codify or extract from the patient’s electronic clinical records [42]. Studies evaluating an ECDSS [43] that applies the explicit criteria in the latest versions of these tools estimate that 67% of the STOPP criteria require additional clinical information vs 31% for Beers. Many studies employ several criteria instead of the complete list; hence, the analysis of prescriptions remains incomplete [44], whereas others [10] use free adaptations of the criteria to facilitate the development of computerized algorithms.

### Strengths and Limitations

Not exporting the data directly from clinical records but from a DCN designed ad hoc could have potentially introduced an information bias regarding illnesses and pharmacological treatment. Given the design of the Multi-PAP trial, health professionals may have prioritized pathologies they considered chronic and were included in the classification employed in the trial instead of registering the total number of patients’ diseases. Something similar may have occurred with topical medication as the research team decided not to include it in the study because it is not associated with significant adverse effects. This bias could have resulted in an overestimation of the prevalence of certain STOPP or START criteria that the research team tried to correct by adjusting the A1P criterion.

The pragmatic design of the study is among our strengths, with patients comprising a representative sample of the community-dwelling young seniors with multimorbidity and polypharmacy, who were interviewed and evaluated by their usual doctors during clinical practice. Overall, this age group has a good quality of life and substantial potential for early interventions.

### Applicability of the Outcome

Using the ECDSS allows for a rapid, complete, and simultaneous evaluation of PIP based on different explicit criteria. This tool can support family doctors when deciding what the best therapeutic choice is for each patient, not only to review the therapeutic plan but also to prescribe new treatments. In any event, even after the information is provided via the ECDSS, the professional must always evaluate it and make the final decision. The decision to continue or discontinue certain treatments must be made based on clinical conditions and within a framework of a doctor-patient shared decision making. The evaluation of this tool in the future in clinical effectiveness and implementation of hybrid design studies will help in integrating them into the clinical electronic records of patients.

## Appendix

Multimedia Appendix 1 Sociodemographic, clinical, and pharmacological characteristics of patients.

Multimedia Appendix 2 Disease List Prevalence according to International Classification in Primary Care.

Multimedia Appendix 3 List of all screening tool of older people's prescriptions, screening tool to alert to right treatment, and Beers criteria.

## Abbreviations

ATC: Anatomical Therapeutic Chemical
ATC-N: Anatomical Therapeutic Chemical classification system-nervous system
BZD: benzodiazepines
COPD: chronic obstructive pulmonary disease
DCN: data collection notebook
ECDSS: electronic clinical decision support system
Multi-PAP: Multimorbidity and Polypharmacy in Primary Care
NSAID: nonsteroidal anti-inflammatory drug
OR: odds ratio
PIP: potentially inappropriate prescribing
PPI: proton pump inhibitor
START: Screening Tool to Alert doctors to Right Treatment
STOPP: Screening Tool of Older Person’s Prescription
V2: version 2
