# Correction: Use of an Electronic Clinical Decision Support System in Primary Care to Assess Inappropriate Polypharmacy in Young Seniors With Multimorbidity: Observational, Descriptive, Cross-Sectional Study

**DOI:** 10.2196/25678

**Published:** 2020-11-19

**Authors:** Eloisa Rogero-Blanco, Juan A Lopez-Rodriguez, Teresa Sanz-Cuesta, Mercedes Aza-Pascual-Salcedo, M Jose Bujalance-Zafra, Isabel Cura-Gonzalez, MultiPAP Group

**Affiliations:** 1 General Ricardos Primary Health Care Centre Madrid Spain; 2 Medical Specialties and Public Health, School of Health Sciences, University Rey Juan Carlos Alcorcón, Madrid Spain; 3 Health Services Research on Chronic Patients Network (REDISSEC) Madrid Spain; 4 Research Support Unit, Primary Care Management Madrid Spain; 5 Dirección Farmacia Atención Primaria Sector Zaragoza III Zaragoza Spain; 6 Dirección Unidad Gestión Clínica Victoria en Málaga, Servicio Andaluz de Salud Málaga Spain; 7 See Acknowledgments

In “Use of an Electronic Clinical Decision Support System in Primary Care to Assess Inappropriate Polypharmacy in Young Seniors With Multimorbidity: Observational, Descriptive, Cross-Sectional Study” (JMIR Med Inform 2020;8(3):e14130) the authors noted one issue.

In the Acknowledgments section of the paper, the statement:

This study was financed by the Fondo de Investigaciones Sanitarias Coordinated project (grant references PI15/00276, PI15/00572, PI15/00996) from the Instituto de Salud Carlos III

has been removed and replaced by the following statement:

This study was funded by National Institute for Health Research ISCIII (Grant numbers PI15/00276 (APT), PI15/00572 (ICG), PI15/00996 (JDPT), RD16/0001/0004 (ICG), RD16/0001/0005 (APT), RD16/0001/0006 (JDPT)) Co-funded by European Regional Development Fund, (ERDF) “A way of shaping Europe”. National Plan I+D+I 2013-2016.

The correction will appear in the online version of the paper on the JMIR Publications website on November 19, 2020, together with the publication of this correction notice. Because this was made after submission to PubMed, PubMed Central, and other full-text repositories, the corrected article has also been resubmitted to those repositories.

